# Surgical treatment of feline meningioma: a single-institution survival analysis

**DOI:** 10.1177/1098612X261421991

**Published:** 2026-01-30

**Authors:** Kathelijn J van Heusden, Lucinda L van Stee, Niels R Blees, Wilhelmina Bergmann, Carles Planas Padrós, Björn P Meij

**Affiliations:** 1 Division Small Animal Surgery, Department of Clinical Sciences, Faculty of Veterinary Medicine, Utrecht University, Utrecht, The Netherlands; 2 Department of Clinical Sciences, Faculty of Veterinary Medicine, Utrecht University, Utrecht, The Netherlands; 3 Division of Pathology, Deparment of Biomolecular Health Sciences, Faculty of Veterinary Medicine, Utrecht University, Utrecht, The Netherlands; 4 Division of Diagnostic Imaging, Faculty of Veterinary Medicine, Utrecht University, Utrecht, The Netherlands

**Keywords:** Intracranial, meningioma, craniotomy, craniectomy, survival time

## Abstract

**Case series summary:**

The aim of this study was to evaluate median survival time (MST), disease-free interval (DFI) and postoperative complications of surgical resection of feline intracranial meningiomas, providing evidence for prognostic counselling for general practitioners. A retrospective review of medical records (2012–2025) identified 17 cats undergoing craniotomy or craniectomy for histologically confirmed meningiomas. Data included patient characteristics, clinical signs, imaging findings, surgical approach, complications, histopathology, recurrence and survival. MRI or CT was used for diagnosis and postoperative monitoring when available. Survival analysis employed Kaplan–Meier and competing risk models; DFI was based on clinical signs or follow-up imaging. A total of 17 cats (median age 11.6 years; 82.4% domestic shorthairs) underwent surgery. The rostrotentorial approach was most common (65%), with minor intraoperative complications in three cases. Postoperative mortality within 4 weeks was 17.6% (3/17). In total, 14 cats survived to discharge. Median follow-up was 622 days. Estimated MST was 1674 days (95% confidence interval [CI] 1395–NE [not estimable]), with 1-, 2-, 3- and 4-year survival rates of 82%, 82%, 82% and 72%, respectively. Median DFI was 377 days (855 days for cats surviving to discharge). Histopathology predominantly included meningothelial and transitional subtypes. Recurrence was detected on MRI in 3/6 cases undergoing follow-up imaging.

**Relevance and novel information:**

Surgical resection of feline meningiomas is a feasible treatment option with excellent outcomes for cats surviving the immediate postoperative period. The study demonstrates a high MST and low recurrence rate among treated cats, with surgery being curative in many cases. These results enable the veterinary general practitioner to more accurately assess the risks and benefits of surgical treatment of feline intracranial meningioma and discuss treatment with owners.

## Plain language summary

Outcomes for cats undergoing surgery for meningiomas at a single clinic

This study evaluated outcomes in cats who underwent surgery to remove intracranial meningiomas, a typically slow-growing brain tumour that is often effectively treated with surgery. The aim was to better understand survival times, the likelihood of tumour recurrence and postoperative complications, allowing veterinarians to offer clearer guidance to owners. Medical records from 17 cats treated between 2012 and 2025 were reviewed. All cats had surgical tumour removal, and the diagnosis was confirmed through histological examination. Most tumours were located in the front part of the brain, and MRI or CT was used for diagnosis and follow-up when available. Surgery proved generally safe: only minor intraoperative complications occurred and 14/17 cats survived to hospital discharge. Three cats (approximately 18%) died within the first 4 weeks after surgery. Among the cats who survived the immediate postoperative period, long-term outcomes were very favourable. The median survival time exceeded 4.5 years, with 82% of cats alive at 1, 2 and 3 years, and 72% still alive at 4 years. The time until tumour recurrence or return of clinical signs varied, but in cats discharged from the hospital, the median disease-free interval was approximately 2.3 years. Follow-up MRI was available in six cats, and tumour regrowth was identified in three. Most tumours were common, less aggressive subtypes. Overall, this study supports surgical removal of meningiomas as an effective and often durable treatment option for affected cats. Those who recover from the initial postoperative period typically experience an excellent long-term prognosis. These findings can help general practitioners counsel owners on the expected benefits and risks of surgical management.

## Introduction

Meningioma is the most common intracranial tumour in cats, accounting for 58% of primary intracranial neoplasms.^1^ These slow-growing tumours arise from arachnoid cap cells and are located outside the brain tissue but within the dura mater.^2^ Histological subtypes include fibrous, transitional, meningothelial, psammomatous, atypical and anaplastic meningiomas.^3^ Older cats (median age 12 years) are mostly affected, and no sex predilection has been described;^4,5^ however, domestic shorthair (DSH) cats may be predisposed.^6^ Clinical signs include altered consciousness, ataxia, circling, blindness, seizures and non-specific signs such as anorexia or lethargy, depending on tumour location and size.^1,2,6,7^

Multiple treatment options for feline intracranial meningiomas are available, with surgical resection being the treatment of choice as it provides a longer survival time (range 685–1345 days)^1,7,8^ than non-surgical treatment (339–515 days).^8,9^ In some cases, it may even be curative. Postoperative complication rates of 54% are described, ranging from minor complications, such as transient hypo- or hyperthermia, a reduced level of consciousness and mydriasis, to major complications, such as seizures, increased intracranial pressure (ICP) and ventricular tachycardia.^10^ Postoperative mortality is reported in 6–17% of cases.^5,10^ Non-surgical treatment options include radiotherapy, with a described median survival time (MST) of 339–515 days, or medical treatment,^8,9^ including the use of corticosteroids and anti-epileptic drugs, with a reported MST of 18 days.^1,11^

This retrospective case series describes the MST and, where possible, the disease-free interval (DFI) for surgical resection of feline meningioma in 17 cats in a single institution. Long-term survival and lack of recurrence in clinical cases in our hospital differed from previously published data, which resulted in the motivation to publish these findings. The goal of this case series is to increase awareness among veterinary general practitioners about the benefits of surgical treatment and to support informed treatment decisions.

## Case series summary

### Materials and methods

#### Study population and data collection

Medical records from a single institution (2012–2025) were reviewed. A total of 17 cats met the inclusion criteria of undergoing curative-intent craniotomy or craniectomy with histologically confirmed meningioma. Retrieved data included signalment (sex and neuter status, body weight, age, breed), clinical history, imaging findings, surgical details, perioperative complications and histological diagnosis. Time to recurrence and overall survival were recorded, with recurrence defined by MRI when available or by return of compatible clinical signs. All records were reviewed by a single investigator (KH).

#### Imaging

All patients included in the study underwent neurological examination by a board-certified surgeon, neurologist and/or internal medicine specialist and imaging by CT and/or MRI. MRI scans were performed with a 1.5 Tesla scanner (Ingenia; Philips Healthcare). Images obtained included T1-weighted, T2-weighted and T1 FLAIR pre- and post-contrast (0.3 ml Dotarem/kg IV) sequences in the transverse and sagittal planes. CT scans were obtained using a 64-slice sliding gantry CT scanner (Somatom Definition AS; Siemens Healthcare) both before and after contrast (Xenetix 350, 2 ml/kg IV). Images were reviewed by residency-trained clinicians under supervision of a board-certified radiologist.

#### Anaesthesia protocol

Anaesthesia protocols were supervised by a board-certified anaesthesiologist in all cases. Routine anaesthesia protocols for craniotomy were conducted as follows: for pre-medication, methadone (0.2–0.3 mg/kg IV/IM) was used. For induction, propofol (1 mg/kg IV) and midazolam (0.1–05 mg/kg IV) were used. Maintenance was conducted using isoflurane inhalation anaesthesia in combination with constant rate infusions (CRIs) of remifentanil (0.2–0.4 µg/kg/min IV), ketamine (10–20 µg/kg/min IV) and/or dexmedetomidine (0.5 µg/kg/h IV). Dexamethasone (0.2 mg/kg IV) was administered according to the surgeon’s preference. If there were signs of increased intracranial pressure (ICP), either hypertonic saline (3–5 ml/kg IV over 15–20 mins) or mannitol (0.5–1 g/kg IV over 15–20 mins) was administered. All patients received cefazoline (20 mg/kg IV) every 90 mins during the procedure. Standard monitoring equipment was used to monitor anaesthetic and physiological parameters every 5 mins, including body temperature, heart rate and rhythm, invasive blood pressure measurement, arterial blood gases and urine production.

#### Surgical technique and histopathology

Patients were positioned in sternal recumbency with the head elevated to promote venous outflow and were immobilised using a vacuum cushion and surgical tape. Aseptic preparation followed a chlorhexidine protocol. All surgeries were performed by one board-certified surgeon (BM). The surgical approach was determined upon review of preoperative medical imaging (MRI/CT) and made through a transfrontal, rostrotentorial, caudotentorial or suboccipital approach, or a combination of these. When performing multiple approaches to remove multiple meningiomas, these were noted as ‘double approaches’. Defects were closed with a fascia graft alone or combined with bone flap replacement secured with a plate and locking screws (LeiLOX Titanium Locking Plate System 1.5; Rita Leibinger Medical). The removed neoplasms were fixed in 10% buffered formalin, embedded in paraffin, sectioned at 4 μm and stained with haematoxylin and eosin for histopathological examination by a board-certified pathologist.

#### Postoperative care

All patients remained in the intensive care unit (ICU) until discharge, supervised by a board-certified intensivist. Postoperative complications were considered ‘minor’ when no surgical intervention was required (anorexia, pain, lethargy, neurological signs, hypertension combined with bradycardia) or ‘major’ if revision surgery was required, if the owner elected euthanasia or when the patient died because of these complications. Postoperative mortality was defined as death within 4 weeks after surgery.

#### Follow-up

Follow-up data were obtained from medical records and a telephone questionnaire. In one case, the owner was not available and the referring veterinarian was contacted. The questionnaire collected data on postoperative recovery, recurrence of clinical signs, follow-up MRI results (if applicable) and date of death. If only the month and year of death were recalled by the owner, the date of death was set for the first day of that month. Follow-up time was defined as the number of days passed since the date of surgery. DFI was defined as the number of days without evidence of recurrence.

#### Statistical analysis

Descriptive statistics were performed using SPSS (IBM). Survival analysis was performed using R v4.5.0 (R Foundation for Statistical Computing), along with the ‘survival’ (v3.8-3),^12^ ‘ggsurvfit’ (v1.1.0),^13^ and ‘cmprsk’ (v2.2)^14^ packages.^15^ Overall survival was evaluated following the Kaplan–Meier method.^16^ Time to event was defined as the time in days from surgery to meningioma-related death or euthanasia. Patients who were lost to follow-up, died from unrelated causes or had not experienced the event by the last follow-up date were considered censored. Survival probabilities and 95% confidence intervals (CIs) at 1–4 years were estimated using the Kaplan–Meier curve. As MST was not reached, restricted mean survival time was calculated. As a result of the low sample size and early censoring, competing risk analysis was additionally performed to reduce the risk of overestimating meningioma-related mortality in the population at risk.

## Results

### Study population characteristics

A total of 17 cats were treated for intracranial meningioma by curative intent tumour resection, with the definitive diagnosis made through histopathological examination. Median age at the time of surgery was 11.6 years (range 6.8–15.1) and median body weight was 4.8 kg (range 3.9–6.6). Of the 17 cats in the study population, there were 14 (82.4%) domestic shorthairs, 2 (11.8%) British Shorthairs and one (5.9%) Maine Coon. There were 12 males (one intact, 11 castrated) and five females (all spayed). Clinical signs included ataxia (71%), lethargy (41%), circling (35%), blindness (18%), head tilt (18%), seizures (12%), nystagmus (6%), paraparesis (12%) and tetraparesis (12%).

### Imaging

**Figure 1:**
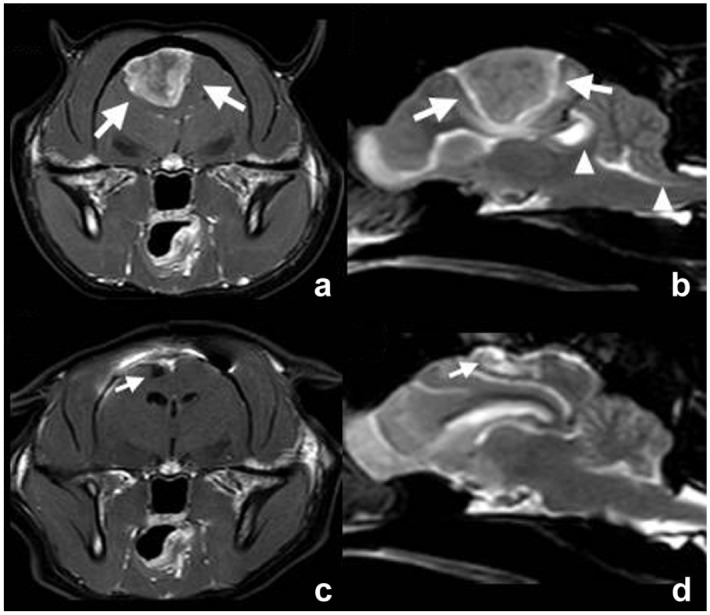
(a,c) Transverse T1-weighted post-contrast images and (b,d) parasagittal T2-weighted images (a,b) before and (c,d) after craniectomy of case 4. In the preoperative images, a large well-defined mass with marked and heterogeneous contrast enhancement and a broad base towards the neurocranium is present in the region of the right parietal lobe (large arrows). Secondary mass effect is present, visible as caudal displacement of the tectum of the midbrain causing rostral deformity of the cerebellum (left arrowhead) and herniation of cerebellar parenchyma through the foramen magnum (right arrowhead). In the postoperative images, the mass and secondary mass effect are no longer present, with only a small fluid-filled cavity present in the region of the craniectomy (small arrows)

Preliminary diagnosis was made using MRI (14 cats) (Figure 1), CT alone (one cat), and CT and MRI (two cats). Median tumour height was 18.7 mm (range 7.0–27.0), median tumour width was 18.0 mm (range 10.0–30.0) and median tumour length was 19.1 mm (range 11.8–31.0). Solitary tumours were seen in 10 (58.8%) cases, with five (29.4%) patients having multifocal lesions. In two (11.8%) cats, imaging was performed at external clinics and lesion extent (solitary or multifocal) was not specifically mentioned in MRI reports. Hyperostosis of the adjacent bone was described in five (29.4%) cases.

### Surgical technique

**Figure 2:**
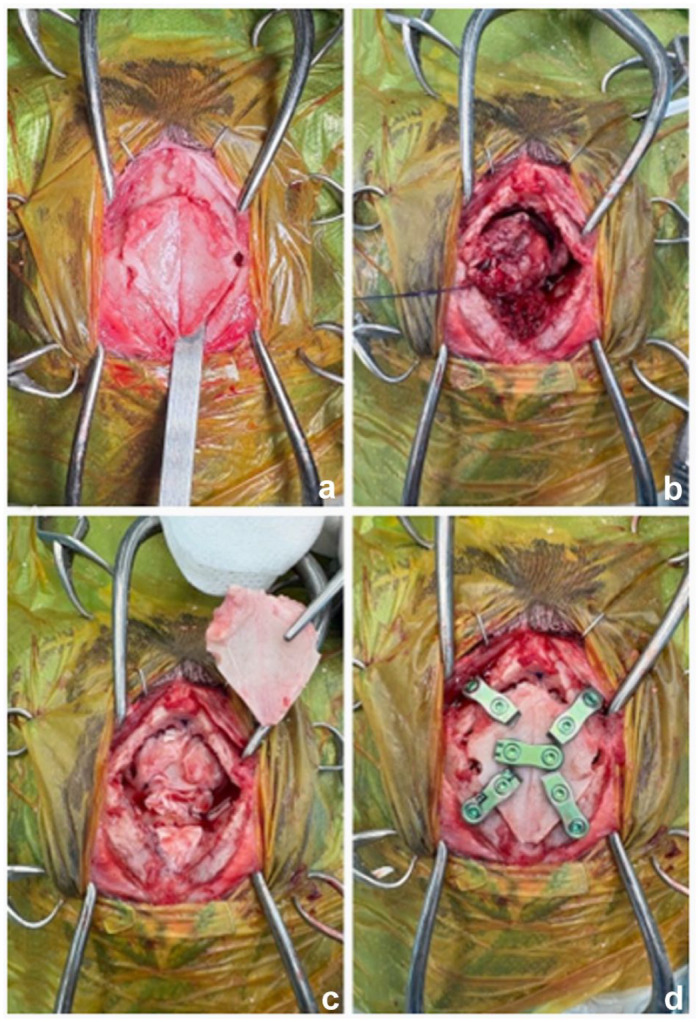
(a) Bilateral transfrontal sinus craniotomy in cat 1 with a meningioma. (b) After removal of the frontal sinus bone flap, the meningioma is carefully extracted. (c) A tensor fasciae latae graft is placed in the craniectomy site on the bottom of the sinus frontalis. (d) The frontal sinus bone flap is replaced and fixed with five two-hole titanium plates and locking screws

A rostrotentorial approach was used in 11/17 (64.7%) cases, a transfrontal approach was used in three (17.6%) cases (Figure 2) and a double approach was used in one (5.9%) case. In two (11.8%) cases, a combined caudotentorial–suboccipital approach was necessary. Minor haemorrhage was seen in three cases. Swelling of the brain was seen in one case. No other intraoperative complications were observed. After removal of the meningioma, the resulting cavity was filled with a sterile gelatine sponge (Spongostan Ethicon) in all but one case. Closure consisted of placing a tensor fasciae latae graft in 12 (70.6%) cases, a masseter fascia graft in two (11.8%) cases or a temporalis fascia graft in one (5.9%) case. In two (11.8%) cases, no fascia graft was placed. Craniotomy was performed in five (29.4%) cases and craniectomy in 12 (70.6%) cases (Table 1).

**Table 1:** Signalment, surgical details, histological diagnosis and follow-up in 17 cats with meningioma

*Case*	*Signalment*				*Tumour details*	*Surgical details*				*Follow*-*up (days)*	
	BW (kg)	Sex	Breed	Age at diagnosis (years)	Diameter (mm) and distribution	Surgical approach	Surgical technique	Closure technique and graft	Histological subtype	ST	DFI
1	4.4	MCa	DS	10.0	18 S	Transfrontal	CO	BF, plate/screws, TFL	Fibrous	17	17
2	6.4	FS	DS	10.6	24 S	Rostrotentorial	CE	TFL	Transitional	111	111
3	5.3	FS	MC	11.6	16 MF	Combined approach	CO	BF, plate/screws, TFL	Meningothelial	377	358
4	4.6	MI	DS	10.5	20 MF	Rostrotentorial	CO	BF, plate/screws, TFL	Fibrous	332	377
5	4.4	MCa	BS	11.6	31 S	Combined approach	CO	BF, plate/screws, TFL	Transitional	622	622
6	4.3	MCa	DS	6.8	17 S	Rostrotentorial	CE	TFL	Fibrous	3	3
7	5.1	FS	DS	12.8	12 S	Rostrotentorial	CE	TFL	Transitional	1519	1519
8	5.4	MCa	DS	13.8	27 MF	Rostrotentorial	CE	TFL	Transitional	1674	79
9	4.6	MCa	DS	15.1	22 MF	Double approach	CE	TFL	Meningothelial	2454	2454
10	5.5	FS	DS	12.0	17 S	Rostrotentorial	CE	TFL	Psammomatous	3	3
11	4.1	FS	DS	12.7	25 S	Transfrontal	CO	BF, sutures	Meningothelial	7	7
12	4.8	MCa	DS	7.5	24 S	Rostrotentorial	CE	MM	Transitional	3173	3173
13	6.0	MCa	DS	11.8	13 S	Rostrotentorial	CE	MM	Psammomatous	1395	1395
14	5.1	MCa	BS	11.5	19 S	Rostrotentorial	CE	TFL	Psammomatous	1966	1966
15	3.9	MCa	DS	8.3	30 NS	Transfrontal	CE	TFL	Meningothelial	2744	2744
16	6.6	MCa	DS	14.2	26 MF	Rostrotentorial	CE	Fat	Meningothelial	1179	1088
17	4.6	MCa	DS	12.5	20 NS	Rostrotentorial	CE	MT	NS	1	1

BF = bone flap; BS = British Shorthair; BW = body weight; CE = craniectomy; CO = craniotomy; DFI = disease-free interval; DS = domestic shorthair; FS = female spayed; MC = Maine Coone; MCa = male castrated; MF = multifocal; MI = male intact; MM = m masseter; MT = m temporalis; NS = non-specified; S = solitary; ST = survival time; TFL = m tensor fasciae latae

### Postoperative mortality and complications

Patients remained in the ICU for a median of 3 days (range 1–10). All patient care was overseen by a board-certified Emergency and Critical Care specialist. Feeding tubes were placed in 5/17 cases to help facilitate delivering oral medication. One-third of patients (5/17, 29.4%) had no postoperative complications. Minor complications occurred in 9/17 (52.9%) cases and included postoperative pain, new-onset seizures, signs of increased ICP (bradycardia, hypertension) and transient blindness. Major complications were seen in 3/17 (17.6%) cases, two of which resulted in euthanasia and one that resulted in spontaneous death. In the first case (case 6), aspiration pneumonia due to inadvertent incorrect feeding tube placement was unresponsive to oxygen and antibiotic therapy, which led to euthanasia. In the second case (case 10), the patient remained severely soporific after surgery and developed bilateral laryngeal paralysis. Thoracic radiographs showed no evidence of aspiration pneumonia, and the diagnosis of bilateral laryngeal paralysis was based on clinical evaluation of laryngeal function. The patient was mechanically ventilated for 2 days without improvement before being euthanased. Another case (case 17) developed Cushing’s reflex (severe hypertension and bradycardia) unresponsive to medical intervention, resulting in respiratory arrest and death. Thus, the postoperative mortality rate was 17.6% (3/17). Post-mortem examination and/or advanced imaging was not performed in deceased cases.

### Survival analysis

**Figure 3:**
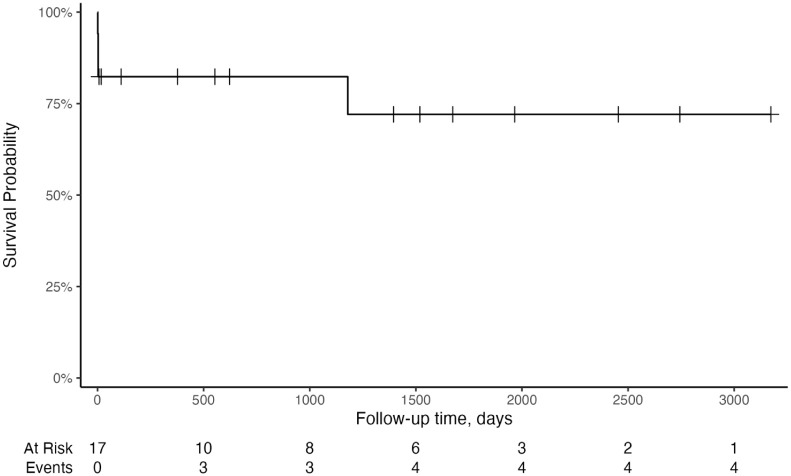
Kaplan–Meier overall survival curve in 17 cats with meningioma who underwent surgical resection

The follow-up time for our patient group was a median of 622 days (range 1–3173). Among patients used for survival analysis, six were still alive at the time of conducting this research. One patient was lost to follow-up. This resulted in an estimated MST of 1674 days (95% CI 1395–NE [not estimable]) or a restricted mean survival of 1265 (95% CI 976–1553) (Table 1, Figure 3). Restricted mean survival until the latest observed moment was 1265 days (95% CI 976–1553). Of the 17 patients, 14 (82.4%) survived to discharge. Of the discharged population (14/17), restricted mean survival until the latest observed moment was 1536 days (95% CI 1442–1629).

The 1-, 2- and 3-year survival rate was 82% (95% CI 66–100). One cat was euthanased in the fourth year because of a return of clinical signs, reducing the 4-year survival rate to 72% (95% CI 51–100) (Table 2).

**Table 2:** Estimated survival rates, 1–4 years postoperatively

Time (days)	n*	Events^†^	Censored	SR (%)	95% CI
365	11	3	3	82	66–100
730	8	0	3	82	66–100
1095	8	0	0	82	66–100
1460	6	1	1	72	51–100

* Number of patients at risk

† Meningioma-related death

CI = confidence interval; SR = survival rate

**Figure 4:**
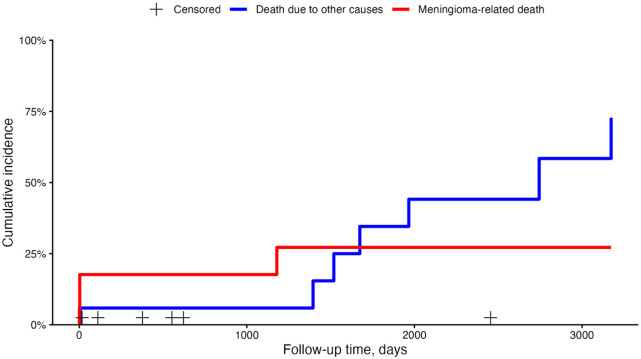
Competing risk analysis in 17 cats with meningioma who underwent surgical resection. + = censored; blue line = death due to other causes; red line = meningioma-related death

Competing risk analysis revealed that the risk of dying from meningioma-related causes did not increase after 1179 days postoperatively, while the risk of dying from unrelated causes continued to increase until the end of our follow-up period (Figure 4).

### Histopathology

**Figure 5:**
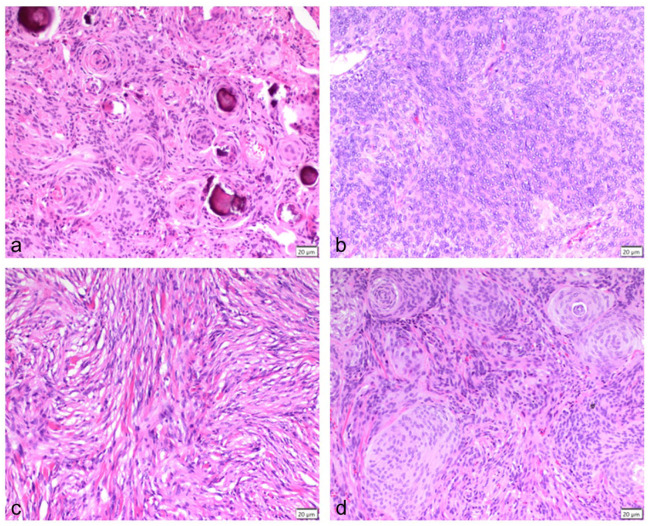
Histological subtypes in cats with meningioma. (a) Psammomatous subtype (case 10). (b) Meningothelial subtype (case 3). (c) Fibrous subtype (case 4). (d) Transitional subtype (case 5)

Histopathological re-evaluation by one of the authors (WB) was available for 14/17 cases. Subtypes included meningothelial (n = 5), transitional (n = 5), fibrous (n = 2) and psammomatous (n = 2) types (Table 1, Figure 5). For three patients, re-evaluation was not possible and the original diagnoses were used: fibrous (n = 1), psammomatous (n = 1) or non-specified (n = 1).

Meningothelial meningiomas comprised polygonal to elongated cells forming sheets, with moderate eosinophilic cytoplasm and centrally placed round nuclei with finely stippled chromatin and a small nucleus. The fibrous subtype was characterised by spindle cells arranged in bundles and streams, with moderate cytoplasm, oval nuclei and intervening mild to moderate collagen. One fibrous meningioma showed focal meningothelial islands with surrounding lymphocytes and plasma cells, and another contained cholesterol clefts with foamy macrophages. Transitional tumours combined spindle cell bundles with meningothelial islands and whorls, occasionally containing psammoma bodies and cholesterol clefts. Psammomatous meningiomas had a similar arrangement, with whorls surrounding abundant psammoma bodies. One psammomatous meningioma also contained extensive cholesterol clefts (Figure 5).

### Recurrence and time to recurrence after surgical treatment

**Figure 6:**
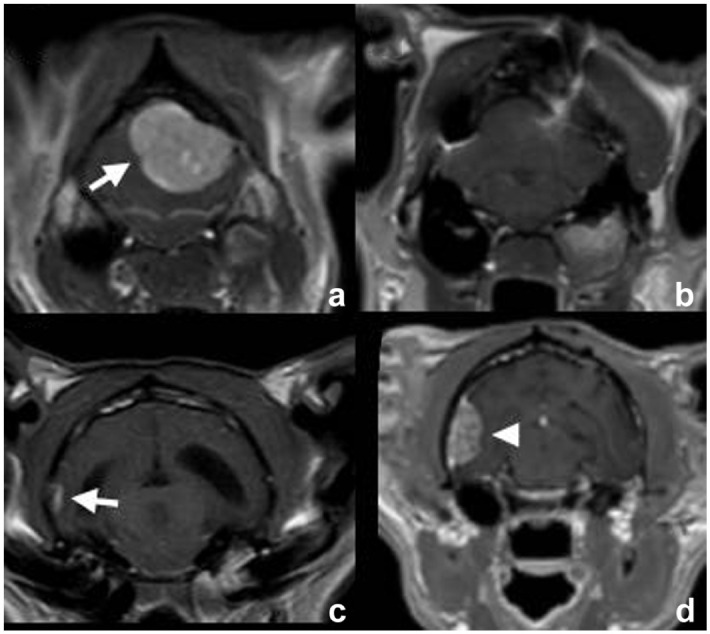
Transverse T1-weighted post-contrast images of case 3 (a,c) before and (b,d) after craniectomy. In the preoperative images, a large well-defined mass with marked and heterogeneous contrast enhancement and a broad base towards the neurocranium is present on the left side of the left occipital lobe (arrow in [a]). In addition, a focal area of increased contrast enhancement is present in the region of the right temporal lobe (arrow in [c]). In the 1-year follow-up MRI, there is no remaining mass in the region of the left occipital lobe (b). However, the lesion in the region of the right temporal lobe is markedly increased in size and appears as a well-defined mass with marked and heterogeneous contrast enhancement (arrowhead in [d])

Median DFI for all cats was 377 days (range 1–3173). DFI for cats who survived until discharge was 855 days (range 7–3173). Follow-up imaging was recommended 6 months postoperatively, but most owners declined imaging in the absence of clinical signs. Consequently, only 6/17 patients underwent repeat MRI. Recurrence of tumour growth on MRI was seen in 3/6 cases. Case 8 showed evidence of recurrent tumour growth on MRI 79 days postoperatively but showed no clinical signs until his death due to unrelated causes 1674 days postoperatively. Case 16 showed recurrence of clinical signs at 1088 days after surgery. This patient had multifocal lesions at the time of presentation. However, because of the lack of follow-up imaging, it is unclear whether this patient showed tumour regrowth at the original resection site or growth of the residual tumour foci. This patient was euthanased 1179 days postoperatively. Case 3 showed residual tumour foci growth on MRI 358 days postoperatively. This was a patient who presented for multifocal lesions at first presentation (Figure 6). The owner elected a second craniotomy and the cat was recovering from this procedure at the time of performing statistical analysis.

## Discussion

This retrospective study gives insight into the long-term outcome and postoperative complication rate after surgical resection of feline intracranial meningioma in 17 cases. The estimated MST of 1674 days exceeds that of previous publications (685–1345 days).^1,5,7,8,10^ In addition, for the cohort that survived to discharge, our MST of 1287 days is superior to that in previous publications (685–1153 days)^1,5,7,10^ and comparable to one study (1345 days).^8^ Competing risk analysis showed that the relative risk of death from meningioma-related causes plateaued after 1179 days, whereas mortality from unrelated causes continued to rise throughout follow-up. This finding may help general practitioners provide more nuanced prognostic counselling, as it highlights that surgery can be curative if cats survive the postoperative period, with patients dying in the long term from unrelated causes rather than from recurrence.

No intraoperative mortality was recorded, consistent with previous reports of 0–1.6%.^5,10^ The postoperative mortality rate of 18.7% was comparable to a previous study (17%)^17^ but was higher than reported in three other studies (7–14.5%).^5,10,18^ All patients who died postoperatively in the present cohort had undergone a rostrotentorial approach, which was the most commonly used approach in our study. Although this likely reflects the predominance of this approach in our cohort rather than a direct causal relationship, a previous study by Morton et al^18^ reported increased odds of complications for rostrotentorial craniotomy, suggesting that further evaluation of approach-related risk is warranted. Of the cases that died in the direct postoperative period in the present cohort, one patient (case 6) died because of aspiration pneumonia due to inadvertent incorrect feeding tube placement. Another patient (case 17) was euthanased because of severely increased ICP resulting in coma. The last patient (case 10) remained severely soporific after surgery and developed bilateral laryngeal paralysis. This patient was mechanically ventilated for 2 days without improvement before being euthanased. In one previous study, severe respiratory compromise occurred in 9.7% of patients undergoing craniotomy, but this was due to aspiration pneumonia, not laryngeal paralysis.^18^ No radiological evidence of aspiration pneumonia was found for case 10. Tumours in the cases that died in the postoperative period were not significantly larger (Table 1), no intraoperative complications were seen and preoperative clinical signs were not significantly more severe or different from other cases within our cohort.

Short-term follow-up was available for all cases, allowing precise identification of the complication rate in the direct postoperative period. Our postoperative complication rate of 52.9% (minor) and 17.6% (major) was comparable to previous literature with comparable sample sizes.^10^ Major complications were seen in 3/17 (17.6%) cases, consisting of severe respiratory issues and increased ICP not responsive to treatment, all resulting in death of the patient. A larger scale retrospective study of the treatment of intracranial tumours in dogs and cats (n = 165) found a complication rate of 52.1% up to 10 days postoperatively; however, only 9% of this study group comprised cats.^18^ A larger scale study by Gordon et al^19^ reported no overall complication rate; however, 31% of cats in this study had anaemia, 24% had no improvement or worsened neurological status, 14% had anorexia and 10% had incisional leakage. In previous studies, anaemia and epilepsy were reported as the most commonly seen postoperative complications.^10,19^ Anaemia was not reported in our study. Haematological testing was not routinely performed during the postoperative period because more recent studies have reported an incidence of hospital-acquired anaemia, with surgical patients and those in ICU settings being at increased risk.^20,21^ Therefore, anaemia may have been present in our cohort but went undetected. Minor intraoperative haemorrhage occurred in three patients in the present study without associated haemodynamic instability or a need for intervention, whereas 4/8 patients in the 1994 study by Gordon et al^19^ required post-haemorrhagic blood transfusions. The reduced perioperative complications reported in the present study may be due to increased surgical expertise and reduced manipulation of brain tissue, as well as advances in preoperative imaging leading to improved surgical planning.

Long-term follow-up was available for all but two cases, one of which was lost to follow-up; the other underwent surgery during the time of statistical analysis. Follow-up MRI was available in six cases and recurrence of tumour growth was seen in 3/6 cases. This constituted either tumour recurrence at the surgical site or progression of residual tumour foci in cases of multiple meningiomas. Tumour recurrence was seen in 8–29% of cases in previous studies, higher than that seen in our study group.^1,5,7,10,19^ In our cohort, DFI for cats who survived until discharge was 855 days (range 7–3173). Of the patients who survived to discharge, 12/14 (70.6%) did not show a recurrence of clinical signs until the end of our follow-up period. One patient was lost to follow-up, and one patient showed a recurrence of clinical signs at 1088 days postoperatively and was euthanased. Multifocal meningiomas were identified in 5/17 (29.4%) of our cases, more than double the 14% previously reported.^11^ Notably, in one case with progression of residual foci, follow-up surgery was elected. This highlights the question of optimal management for multiple meningiomas: whether to pursue early surgical intervention of (multiple) smaller foci, potentially necessitating a double approach during initial surgery (case 9), or careful monitoring for clinical signs during follow-up, potentially necessitating a second surgery if progression of residual foci is observed (case 3).

Possible prognostic factors identified in previous studies include tumour location, with supratentorial cases showing less favourable outcomes.^17^ The current cohort includes two supratentorial tumours (cases 3 and 9), both of which did not show major complications after surgery and showed a postoperative survival of 377 and 2454 days, respectively. However, this study included only cats who underwent surgical treatment, excluding those deemed unsuitable candidates because of tumour location, comorbidities or owner limitations, which may have introduced selection bias. The severity of clinical signs at presentation did not seem to be related to worse outcomes in our study group; similar results were seen in previous studies.^7,10^ Future studies with a larger cohort are needed to further define prognostic factors.

Histological distribution in our study was broadly consistent with previous reports,^5,11^ with meningothelial and transitional subtypes most common. Fibrous and psammomatous types were under-represented. Limited postoperative MRI follow-up also affects recurrence detection and DFI accuracy, although the lack of return of clinical signs in our case series may be seen as a favourable outcome. Dural biopsy in the vicinity of the meningioma tumour mass may allow assessment of completeness of resection but this study did not focus on the relationship between complete histological resection of tumour and the recurrence rate because of lack of data on dural biopsies. Future study with larger sample sizes and data on dural biopsies may benefit decision-making in postoperative adjunctive therapy and follow-up protocols.

The limitations in our study include the retrospective design and the small number of cases in our study population. Medical records may not have been complete or owner recollection may have been lacking, leading to information bias. Follow-up MRI was not available for all cases, and post-mortem examination or additional imaging was not performed in deceased patients, limiting the ability to assess tumour recurrence and precise cause of death. Haematological and biochemical testing were not consistently performed preoperatively, preventing comprehensive evaluation of pre-existing conditions that may have influenced outcomes. Information on postoperative ongoing medical treatment was not included in data selection and may provide further insight into long-term neurological outcome in future studies. A large-scale, prospective study is needed to gain further confirmation of our results. However, the results show promising survival times and lack of recurrence of clinical signs, providing a valuable foundation for more accurate assessment of risks and benefits of surgical treatment of feline intracranial meningioma.

## Conclusions

Surgical treatment for feline meningioma is a feasible treatment option with excellent outcomes if the patient survives the immediate postoperative period. Given the high MST and low recurrence rate in our study group, surgical intervention should be considered even in older cats, regardless of severity of clinical signs at presentation. These results enable the veterinary general practitioner to more accurately assess the risks and benefits of surgical treatment of feline intracranial meningioma and discuss treatment options with owners.
